# Retraction: Application of in-silico drug discovery techniques to discover a novel hit for target-specific inhibition of SARS-CoV-2 Mpro’s revealed allosteric binding with MAO-B receptor: A theoretical study to find a cure for post-covid neurological disorder

**DOI:** 10.1371/journal.pone.0344338

**Published:** 2026-03-09

**Authors:** The PLOS One Editors

Following publication, concerns were raised about this article [1]. In editorial follow-up, the *PLOS One* Editors identified:

- Concerns about the article’s peer review.
- Concerns about compliance with PLOS policies on authorship and on competing interests.
- Concerns about the molecular docking (MD) simulation data reported in Fig 15.

Specifically, the three replicate simulations in 15i-iv appear similar to each other despite different average quantitative values for the same MD replicas represented in Table 3.

The corresponding author RDJ indicated that the original replicas appeared similar due to system stability, while quantitative differences resulted from using different velocity seeds. RDJ reported that the original simulation files were unavailable, but provided data from three new independent simulations. In the absence of the original files, it could not be clarified whether the findings reflect intrinsic system properties or artefacts of initial conditions; the *PLOS One* Editors consider that the repeat simulation data were not sufficient to resolve this issue and raised further concerns about the applied methodology and support for the published results.

PLOS regrets that the issues were not addressed prior to the article’s publication.

In light of the above concerns, the *PLOS One* Editors retract this article.

It was additionally identified in editorial follow-up that a production error resulted in duplication of the same figure as Figs 15 and 16, and omission of the correct image for Fig 17; consequently, there is a mismatch of figure content, captions and figure numbers for Figs 16 and 17 in the published article. The publisher apologizes for this error, which did not contribute to the decision to retract based on the issues described in the notice above.

RDJ did not agree with retraction. MEAZ, SAAH, AAAM, AS, VHM, RGI, VDR, NMG, SR, PNK, and PVB either did not respond directly or could not be reached
